# Retraction Note to: Schnurri-3 regulates BMP9-induced osteogenic differentiation and angiogenesis of human amniotic mesenchymal stem cells through Runx2 and VEGF

**DOI:** 10.1038/s41419-021-04443-8

**Published:** 2021-12-09

**Authors:** Yuwan Li, Ziming Liu, Yaping Tang, Wei Feng, Chen Zhao, Junyi Liao, Chengmin Zhang, Hong Chen, Youliang Ren, Shiwu Dong, Yi Liu, Ning Hu, Wei Huang

**Affiliations:** 1 Department of Orthopaedics, the First Affiliated Hospital of Chongqing Medical University, Chongqing, 400016 China; 2 Institute of Sports Medicine of China, Peking University Third Hospital, 100191 Beijing, China; 3 Department of Stomatology, Daping Hospital, Army Medical University (Third Military Medical University), Chongqing, 400042 China; 4 Laboratory of Skeletal Development and Regeneration, School of Life Sciences, Chongqing Medical University, Chongqing, 400016 China; 5 Department of Orthopaedics, Southwest Hospital, Army Medical University (Third Military Medical University), Chongqing, 400038 China; 6 Department of Orthopaedics, the Second Affiliated Hospital of Chongqing Medical University, Chongqing, 400000 China; 7 Department of Orthopaedics, the First Affiliated Hospital of Zunyi Medical University, Zunyi, 563000 China

**Keywords:** Mesenchymal stem cells, Stem-cell differentiation

Retraction Note to: *Cell Death & Disease* 10.1038/s41419-020-2279-5 published online 29 January 2020

The Editors-in-Chief have retracted this article due to concerns raised about Figs. 5 and 6. In Fig. 5D, it appears that the partially enlarged image in the sim-shn3 group is not a part of the low-magnification image. Additionally, the images of the BMP9 group detecting CD31 and the BMP9 + Shn3 group detecting MECN are very similar. It appears that the images of these two different groups are from a unified tissue specimen. In Fig. 6E, the images of RFP, BMP9 and sim-Shn3 groups appear to derive from the same culture cells. The results of this article are therefore unreliable.

Author Wei Huang stated on behalf of all co-authors that they agree to this retraction.

